# Pediatric Abdominal Pain: Boba Tea and Computed Tomography Findings: Case Report

**DOI:** 10.5811/cpcem.47218

**Published:** 2025-10-21

**Authors:** Jesse Ewaldt, James Waymack, Sharon Kim

**Affiliations:** Southern Illinois University School of Medicine, Department of Emergency Medicine, Springfield, Illinois

**Keywords:** bubble tea, boba tea, computed tomography (CT), case report

## Abstract

**Introduction:**

Discovery of pearl-like, radiopaque foreign bodies is not widely documented in the literature. In this report, we describe an unusual radiological finding of bubble tea pearls (small, chewy spheres derived from cassava starch) on computed tomography (CT) from an increasingly popular drink among adolescents.

**Case Report:**

An 11-year-old female presented to the emergency department with severe abdominal pain. Physical examination revealed generalized abdominal tenderness, with increased pain in the right lower quadrant. The patient’s history was concerning for acute appendicitis. Laboratory results were unremarkable, and ultrasound was inconclusive for suspected appendicitis. A contrast-enhanced CT of the abdomen found several ingested radiopaque densities within the stomach. Further toxicology testing was negative or within normal limits. It was later found that the patient had consumed bubble tea earlier in the day. The patient was admitted for monitoring, and symptoms resolved spontaneously the following morning.

**Conclusion:**

When pearl-like, radiopaque densities are found in the abdomen, bubble tea could be considered as a possible etiology to prevent unnecessary workup and exposure to radiation for pediatric patients.

## INTRODUCTION

Using computed tomography (CT) to evaluate pediatric patients for abdominal pain in the emergency department (ED) is not routinely recommended due to the increased risk of radiation exposure.^1^ However, CT may be necessary in the evaluation of certain diagnoses such as appendicitis following appropriate clinical decision-making and considering resource availability. Here we discuss an incidental finding of ingested foreign bodies on CT, later identified as bubble tea pearls, in a patient presenting with abdominal pain.

Bubble tea, also known as boba tea or pearl milk tea, is a popular Taiwanese beverage that traditionally consists of milk, tea, and tapioca balls (boba pearls). This drink is highly customizable, contributing to its rise in popularity. Tapioca is a starch that is extracted from cassava roots and often mixed with sweet potato starch to give the final bubble pearls a more durable and chewy structure. Despite being part of a beverage, the bubble pearls themselves are meant to be chewed before swallowing to properly digest their dense, gelatinous nature.

Bubble tea pearls being identified on abdominal CT is not well documented in the literature. Upon review, only two such articles were found of an abdominal CT demonstrating bubble tea pearls.^2^,^3^ This finding may have implications on patient care, and thus may be important for emergency physicians to recognize.

## CASE REPORT

An 11-year-old female patient with no past medical history presented to the ED with eight hours of abdominal pain rated 10/10 in severity. The pain was described as sharp in nature, intermittent, and worse in the right lower quadrant with radiation to the left lower quadrant and epigastrium. The patient denied fever, vomiting, and diarrhea but did endorse nausea with her symptoms. There were no alleviating or aggravating factors. Presenting vital signs were within expected range for age: blood pressure 119/73 millimeters of mercury, heart rate 72 beats per minute, respiratory rate 18 breaths per minute, oral temperature 36.6 °Celsius, oxygen saturation 100% on room air, and weight 47 kilograms.

**Image 1:**
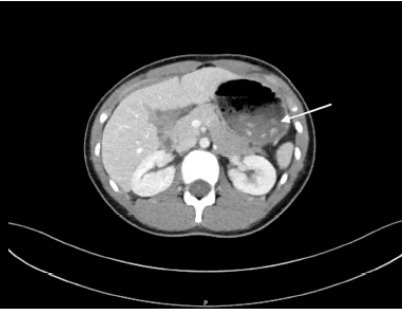
Axial computed tomography with arrow demonstrating radiopaque foreign bodies within the stomach.

**Image 2:**
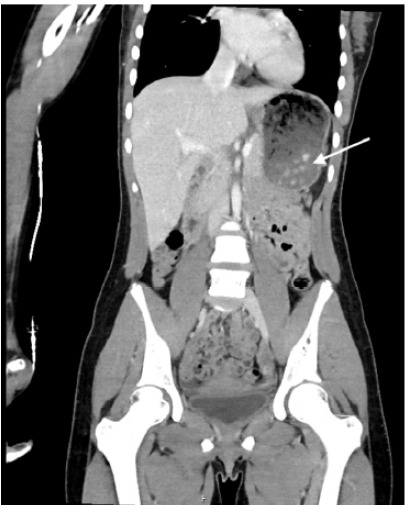
Coronal computed tomography with arrow demonstrating radiopaque foreign bodies within the stomach.

The patient’s physical exam was significant for abdominal tenderness that was generalized but subjectively worse in the right lower quadrant. There was no rebound, guarding or Rosving’s sign present. The patient did have pain when jumping at bedside in the right lower quadrant. The patient underwent evaluation for appendicitis including laboratory blood testing and an ultrasound of the appendix. Initial laboratory values are listed in the Table. The patient’s urine was collected via clean catch which was negative for glucose, ketones, blood, nitrites, leukocytes, and bacteria. The urine sample had less than one white blood cell per high power field (wbc/hpf) (reference range: 0–6 wbc/hpf). Abdominal ultrasound was significant for a non-visualized appendix. The patient continued to have persistent abdominal pain in the right lower quadrant and acute appendicitis or other concerning abdominal pathology were still strongly considered. While magnetic resonance imaging may have been the test of choice in this clinical situation, that imaging modality was unavailable. Following local practice guidelines and resource availability, a CT scan of the abdomen with intravenous contrast was obtained. The CT was significant for a fluid distended stomach with several ingested radiopaque densities within the stomach and a non-visualized appendix (Image 1 and Image 2).

*CPC-EM Capsule*

What do we already know about this clinical entity?

*Computed tomography (CT) is a frequently used diagnostic modality. Bubble tea is an increasingly popular consumed food item*.

What makes this presentation of disease reportable?

*The radiographic appearance of bubble tea is not well documented. This case shows radiopaque densities on CT after bubble tea ingestion, mimicking pathology*.

What is the major learning point?

*Consumption of bubble tea without properly chewing or digesting may lead to pearl-like radiopaque densities appearing on CT*.

How might this improve emergency medicine practice?

*Emergency medicine physicians should consider bubble tea as a possible etiology of radiopaque foreign bodies to prevent unnecessary workup or misdiagnosis*.

A consideration for possible ingestion or overdose was suggested by the radiologist. Workup was amended to include an acetaminophen, salicylate, iron levels, and urine drug screen. Toxicology testing returned negative or within normal limits. Upon re-evaluation of the patient’s history, she denied intentional ingestion of pills or foreign objects. Her mother endorsed that the patient had ingested bubble tea while shopping earlier that evening. The patient was admitted to the pediatric hospitalist service for monitoring and serial abdominal examinations. The patient’s symptoms resolved spontaneously without intervention by the following morning, and she was discharged home with primary follow-up.

## DISCUSSION

This case highlights the finding of bubble (“boba”) tea as a possible cause of radiopaque foreign bodies within the stomach. This can be an important historical finding as it may lead to unnecessary workup, procedures, hospitalization or medication administration that would not be beneficial or could even be harmful to patients. Therefore, it is important that emergency medicine physicians be aware of such historical ingestions when considering the differential diagnosis for acute abdominal pain. As bubble tea has become more popular in recent years, this may be a more common incidental finding in the future.^4^

Bubble tea is comprised of pearls or ‘boba’ that are made from a collection of starches, brown sugar and water that are rolled together in small balls and boiled into a gummy and chewy substance for ingestion. The main starch is tapioca, derived from the cassava root, which is sometimes mixed with potato starch to provide a firm finish.^5^ It is not well understood how this food item shows up as radiopaque foreign bodies within the stomach. Radiopacity is an intrinsic feature of an object that depends on its ability to absorb (attenuate) or scatter X-ray photons.^6^ Further complicating this topic, radiographic visibility of an object can depend not only on its size and radiopacity but also on its anatomic location, the patient’s body habitus, and the surrounding anatomic structures.^7^ Literature review demonstrates only two documented cases of similar occurrence on CT scan.^2^,^3^

The presence of unexpected intraluminal hyperdensities can potentially cause erroneous interpretation of images and consideration for other differential diagnoses such as foreign body or toxicologic ingestion.^6^ The mechanism by which bubble tea forms a hyperdense body may not be obvious but could be explained by general radiologic terms. It may be beneficial for radiologists as well as emergency providers to be aware of this finding as it may change management of patient care. In the case presented, additional laboratory testing and an unnecessary hospital admission.

## CONCLUSION

Bubble tea pearls may be seen as radiopaque foreign bodies on abdominal CT imaging. Physicians should be aware of this potential finding when evaluating patients for abdominal pain or gastrointestinal complaints to help determine if further workup is necessary.

## Figures and Tables

**Table:** Initial laboratory and comprehensive metabolic panel values.

Test	Patient Values	Reference Range	Units
White blood cell count	10.51	4.5–13.5	cells/μL
Hemoglobin	12.6	11.5–15.5	g/dL
Platelet count	371	150 – 400	×10^3^/μL
Sedimentation rate	4	0–20	mm/hr
C-reactive protein	<0.29	<0.8	mg/dL
Lipase	26	13–75	U/L
Sodium	141	136–145	mEq/L
Potassium	3.8	3.5–5.1	mEq/L
Chloride	111	98–107	mEq/L
Bicarbonate	26.4	21–32	mEq/L
Anion gap	3.6	5–15	mEq/L
Blood Urea Nitrogen	8	7–18	mg/dL
Creatinine	0.68	0.55–1.02	mg/dL
Alkaline Phosphatase	158	178–526	U/L
Aspartate transaminase	13	15–37	U/L
Alanine transaminase	17	13–56	U/L
Total bilirubin	0.8	0.2–1	mg/dL

Abbreviations: cells/μL, cells per microliter; g/dL, grams (g) per deciliter (dL); ×10^3^/L, platelets per μL; mm/hr, millimeters per hour; mg/dL, milligrams (mg) per dL; U/L, units (U) per liter (L); mEq/L, milliequivalents per liter.
